# Functional and Oncologic Outcomes in Single-Kidney Patients Treated with Robot-Assisted Partial Nephrectomy for Renal Tumors: Results from a Prospectively Maintained Dataset of a Single Tertiary Referral Center

**DOI:** 10.3390/cancers17121978

**Published:** 2025-06-13

**Authors:** Antonio Andrea Grosso, Luca Lambertini, Fabrizio Di Maida, Giulia Carli, Pedro Ramos, Alessandro Sandulli, Vincenzo Salamone, Francesca Conte, Filippo Lipparini, Elena Ciaralli, Daniele Paganelli, Sofia Giudici, Rino Oriti, Riccardo Fantechi, Matteo Salvi, Gianni Vittori, Maria Rosaria Raspollini, Gabriella Nesi, Andrea Minervini, Andrea Mari

**Affiliations:** 1 Department of Experimental and Clinical Medicine, University of Florence, 50121 Firenze, Italyfabriziodimaida@unifi.it (F.D.M.); elena.ciaralli@unifi.it (E.C.);; 2 Unit of Oncologic, Minimally-Invasive Robotic Urology and Andrology, Careggi Hospital, 50134 Florence, Italy; 3 School of Medicine, University of Minho, 4710-057 Braga, Portugal; 4 Life and Health Sciences Research Institute (ICVS), School of Medicine, University of Minho, 4710-057 Braga, Portugal; 5 Department of Urology, Centro Hosspitalar São João, 4200-319 Porto, Portugal; 6 Department of Human Pathology and Oncology, University of Florence Careggi Hospital, 50134 Florence, Italygabriella.nesi@unifi.it (G.N.)

**Keywords:** partial nephrectomy, robotic surgery, RCC, single kidney

## Abstract

**Background**: Renal tumors in solitary kidneys require treatments that optimize both oncological and functional outcomes. Robot-assisted partial nephrectomy (RAPN) offers a balance between these needs and reduced morbidity. This study investigates the oncologic and functional outcomes of RAPN in solitary-kidney patients. **Methods**: We analyzed data from 1852 patients with cT1-T4N0M0 renal cell carcinoma treated by RAPN from January 2018 to June 2022. The cohort included patients with solitary kidneys based on preoperative characteristics, tumor staging and perioperative outcomes using the Trifecta criteria. **Results**: Of the study participants, 39 had solitary kidneys. Fifteen patients (38.6%) had an ASA score > 2, indicating a higher preoperative risk. The median PADUA score was 7 (IQR 8–9). Moreover, 28 (71.8%) patients had a chronic kidney disease stage > 2. Trifecta success was achieved in 26 (66.6%) of the cases. During a median follow-up of 36 months, tumor recurrence was observed in 12 patients (30.7%), with local recurrences in 4 (10.2%) and systemic recurrences in 8 (20.5%). A higher ASA score and global ischemic clamping were independent predictors of renal function decline at the third postoperative day and Trifecta failure. Only a higher ASA score significantly predicted a significant long-term decline in renal function. Nucleolar grade at pathological stage was the only factor significantly associated with tumor recurrence. **Conclusions**: RAPN is as an effective treatment for renal tumors in solitary kidneys, balancing oncological control and renal function preservation. Global ischemia and patient physical status are the most important factors influencing outcomes and highlight the importance of patient selection and tailored surgical strategies.

## Simple Summary

The presence of renal tumors in solitary kidneys represents a unique challenge, necessitating a delicate balance between achieving optimal oncological control and preserving renal function. In this scenario, nephron-sparing surgery, particularly robot-assisted partial nephrectomy (RAPN), has an important role mostly in high-risk patients, in whom it is mandatory to improve precision, reducing the potential morbidity connected to the surgery. The aim of this article is to assess the perioperative, functional and oncologic outcomes of solitary-kidney patients with renal tumors treated with RAPN, relying on a prospectively enrolled cohort from a very-high-volume center.

## 1. Introduction

The presence of renal tumors in solitary kidneys represents a critical challenge, necessitating a delicate balance between achieving optimal oncological control and preserving renal function [1]. Nephron-sparing surgery (NSS), particularly partial nephrectomy, remains the gold standard for managing renal masses in this context, ensuring maximal preservation of functional renal parenchyma while addressing oncologic objectives [2]. The evolution of minimally invasive surgical techniques, including robot-assisted partial nephrectomy (RAPN), has further expanded the feasibility and applicability of NSS in these high-risk patients [3].

**Figure 1:**
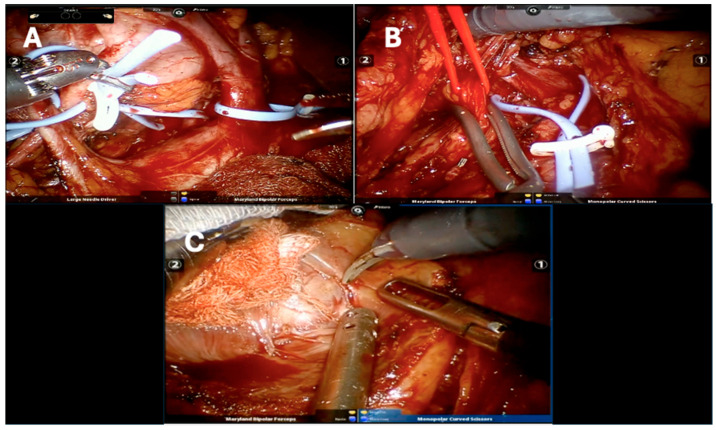
Surgical phases of robot-assisted partial nephrectomy: (**A**) pedicle isolation; (**B**) arterial clamping; (**C**) tumor excision.

The adoption of RAPN in solitary-kidney patients is driven by its precision, reduced morbidity and potential for minimizing ischemia time, factors critical to maintaining renal function postoperatively [4,5,6] (Figure 1).

Although retrospective studies and multi-institutional reviews have provided valuable insights into RAPN’s efficacy, there remains a need for prospective data that comprehensively evaluate both oncological outcomes and functional preservation [7,8,9,10]. Solitary-kidney patients provide a unique model for studying these outcomes, as their renal reserve depends entirely on the preserved kidney, heightening the stakes of surgical intervention.

The aim of the present study was to assess the perioperative, functional and oncologic outcomes of solitary-kidney patients with renal tumors treated with RAPN, relying on a prospectively enrolled cohort from a very-high-volume center.

## 2. Materials and Methods

### 2.1. Patients, Dataset and Selection

We reviewed the clinical data of 2123 consecutive prospectively recorded patients treated RAPN for cT1-T4N0M0 renal cell carcinoma (RCC) from January 2018 to March 2023. Only solitary-kidney patients were considered eligible for analysis. Preoperative features of patients, including age, gender, body mass index (BMI) and comorbidity status, assessed by the Charlson comorbidity index (CCI) and the American Society of Anesthesiologists (ASA) physical status classification system, were collected. All patients underwent preoperative staging using contrast-enhanced multiphasic imaging of the abdomen and chest, performed either by computed tomography (CT) or magnetic resonance imaging (MRI).

All patients were scored according to the preoperative aspects and dimensions used for an anatomical (PADUA) score [11] and radius–exophycity–nearness–anterior–location (RENAL) classification of renal tumors [12]. Tumor stage was classified according to the TNM criteria [13] and nucleolar grading according to the most recent International Society of Urological Pathology (ISUP) grading recommendation by two expert uropathologists (M.R.R., G.N.) [14]. Histopathology was reviewed according to the WHO 2016 classification. Regarding perioperative features, complications were graded according to the Clavien–Dindo (CD) classification [15]. The presence of ink at the resected margins on gross assessment, confirmed by microscopic extension of malignant cells at the stained margins on final histopathological examination, was reported as a PSM. Intraoperative frozen section analysis was not performed.

### 2.2. Follow-Up

Patients with <24 months of follow-up were excluded from the analysis. The follow-up schedule included blood analysis for renal function assessment, chest X-ray and ultrasonography of the abdomen followed by the alternating use of CT scan performed every three months from the first to the second postoperative year, every six months until the fifth postoperative year and then annually according to the risk profile, as recommended by the European Association of Urology (EAU) guidelines. Tumor recurrence was defined as a new lesion demonstrated by imaging, with definitive diagnosis confirmed only after histopathological examination. Furthermore, disease recurrence was dichotomized into local (LR) and systemic (SR) recurrence. LR was defined as any recurrence localized in the ipsilateral kidney. SR was defined as any other recurrence away from the ipsilateral kidney, including the ipsi- or contralateral retroperitoneal lymph nodes or distant organs.

### 2.3. Outcomes

The main endpoint of the study was to assess the safety and efficacy of RAPN estimated by Trifecta achievement. Trifecta was defined as the contemporary accomplishment of the following outcomes: (1) absence of Clavien–Dindo > 2 complication; negative surgical margins; and decline in estimated glomerular filtration rate (eGFR) at 3rd postoperative day < 30% from the baseline. Predictors of Trifecta failure were also assessed. Secondary endpoints were recurrence rate and ∆ last follow-up creatinine serum level, defined as the difference between last follow-up and baseline creatinine serum levels.

### 2.4. Statistical Analysis

For statistical purposes, independent variables included all patient- and tumor-related data available in our institutional RCC database. First, descriptive statistics were obtained, reporting medians (and interquartile ranges, IQR) for continuous variables and frequencies and proportions for categorical variables, as appropriate. Sensitivity analysis was performed to compare clinical and surgical features among patients reaching vs. not reaching Trifecta.

Univariable logistic analysis was performed to evaluate factors significantly associated with a specific outcome. Multivariable logistic regression analyses were fitted to explore clinical and surgical predictors of Δ% eGFR loss > 30% at the 3rd postoperative day and Trifecta failure, while Cox regression analyses were used to assess Δ% eGFR loss > 30% and disease recurrence at last follow-up. All statistical analyses were performed using R software (version 4.3.2). All tests were two-sided with a significance set at *p* < 0.05.

## 3. Results

**Figure 2:**
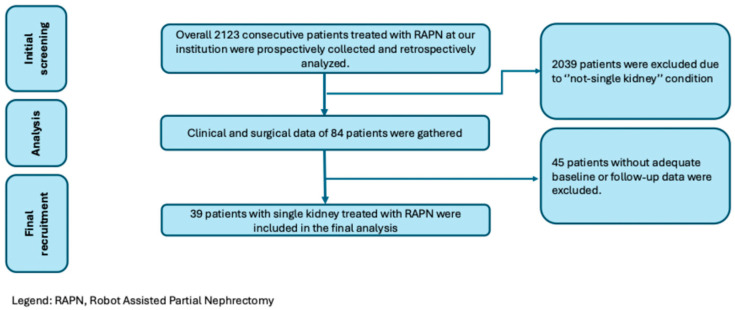
Inclusion criteria.

Overall, 2123 patients treated with RAPN were gathered; of those, 2084 did not meet the inclusion criteria and were excluded, while 39 patients who underwent RAPN for renal tumors in a solitary kidney were included (Figure 2).

The characteristics of the patients at baseline are summarized in Table 1. The median age was 65 (IQR 56–71) years, and the median CCI was 2 (IQR 4–5). Thirty-five (89.8%) patients had an ASA score >II. Solitary kidney was due to previous surgical nephrectomy in 21 (53.8%), congenital anomalies in 10 (25.6%) and functional reasons in 8 (20.6%) patients. Median PADUA and RENAL scores were 7 (IQR 8–9) and 5 (IQR 6–7), respectively, while 33 (84.5%) cases were cT1 stage.

The perioperative features are summarized in Table 2.

The surgical approach was predominantly transperitoneal (74.4%), with a median operative time of 87 (IQR 115–160) minutes. Clamping techniques were employed in 79.4% of cases, with global clamping accounting for 53.8% and selective clamping for 25.6%. The median ischemia time was 12 (IQR 7–18) minutes, and eight (20.6%) cases were completed without clamping. The median operative time was 300 (IQR 250–450) minutes. Overall, 25 (64.1%) patients experienced postoperative complications: 12 (30.7%) received transfusions for bleeding (Clavien grade 2 complication); 2 (2.6%) patients were treated with superselective embolization for significant bleeding (Clavien grade 3a complication) and 1 (5.1%) patient was treated with ureteral stenting for a urinary fistula (Clavien grade 3b complication). Patients had a median Δ creatinine serum level of 1.5 (IQR 1.1–1.9) mg/dL and a Δ eGFR of 20 (IQR 5–45) % loss at the third postoperative day. Overall, 13 (33.3%) patients had a Δ eGFR loss > 30% at the third postoperative day.

**Figure 3:**
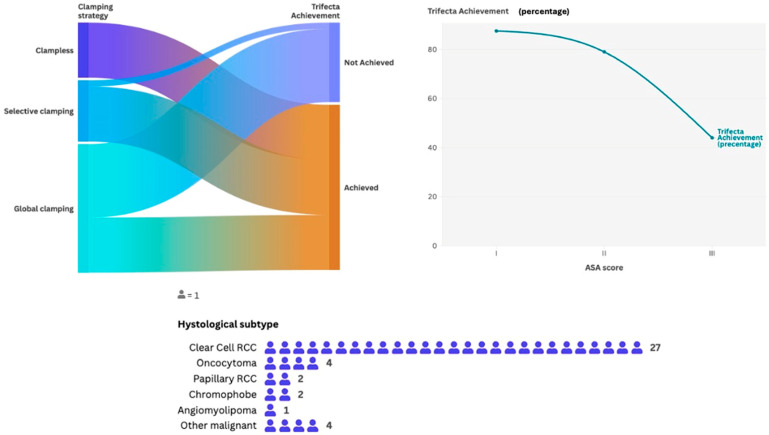
Trifecta achievement.

Positive surgical margins were detected in six (15.3%) cases. Overall, Trifecta was achieved in 24 (61.5%) patients (Figure 3). The median drainage time was 2 (IQR 2–3) days, and the median hospital stay was 4 (IQR 3–5) days.

Pathology features are summarized in Table 3. Malignant histologies were identified in 87.3% of cases, with clear cell renal cell carcinoma (ccRCC) being the predominant subtype, accounting for 69.2% (n = 27). Less frequent malignant variants included papillary RCC (n = 2, 5.1%), chromophobe RCC (n = 2, 5.1%) and other rare malignant histologies (n = 3, 7.9%). Benign histologies were observed in 12.8% of patients, most commonly oncocytoma (n = 4, 10.2%), followed by angiomyolipoma (n = 1, 2.5%).

The median pathological tumor diameter was 30 (IQR 20–49) mm. In terms of nucleolar grade distribution, the majority of tumors were graded as ISUP/WHO grade 2 (47.1%), while grade 1, 3 and 4 were each observed in 4 (11.7%), 9 (26.4%) and 5 (14.8%) cases.

Pathological staging revealed that over half of the tumors were classified as pT1a (n = 20, 51.2%). Tumors staged as pT1b and pT2 were identified in 28.2% (n = 11) and 5.1% (n = 2) of patients, respectively. Notably, locally advanced disease (≥pT3) was detected in 17.8% of cases, with five patients (14.7%) staged as pT3a and one (2.9%) as pT3b. Features of aggressive tumor biology were also documented. Tumor necrosis and sarcomatoid differentiation were each reported in 7% (n = 3) of specimens.

Median overall follow-up was 36 (IQR 26–48) months. Tumor recurrence was detected in 12 (30.7%) patients, being local and systemic in 4 (10.2%) and 8 (20.5%), respectively. Cancer-specific and overall survival reached 100% and 97.4%. Median Δ creatinine at last follow-up from baseline was 0.8 (IQR 0.6–1.4) mg/dL, and Δ% eGFR loss was 10 (IQR 7–30) %. Overall, seven (17.9%) patients had a Δ eGFR loss >30% at last follow-up.

Sensitivity analysis results comparing clinical and surgical data of patients reaching vs. not reaching Trifecta are reported in Table 4. Patients not reaching Trifecta had a significantly higher ASA score compared to those who reached Trifecta (ASA 3: 69.4% vs. 15.5%, *p* < 0.001). Patients not reaching Trifecta had a significantly higher global clamping rate compared to those who reached Trifecta (92.3% vs. 34.6%, *p* < 0.001). Further analysis depicting patients with/without recurrence and complication, stratified by tumor stage and clamping type, are reported in Supplementary Table S1.

Univariable logistic regression showed that ASA score and type of clamping were the clinical and surgical features significantly associated with the three outcomes evaluated.

In multivariable logistic regression analysis (Table 5), ASA score (>II vs. <II) was an independent predictive factor of Δ% eGFR loss >30% at the third postoperative day (OR 1.19, 95%CI 1.13–4.21, *p* = 0.002), Trifecta failure (OR 1.24, 95%CI 1.10–3.87, *p* = 0.001) and Δ% eGFR loss >30% at last follow-up (OR 2.21, 95%CI 1.47–6.81, *p* < 0.001); conversely, global clamping (compared to either selective or clampless) was an independent predictive factor of Δ% eGFR loss >30% at the third postoperative day (OR 1.82, 95%CI 1.43–2.31, *p* < 0.0001) and of Trifecta failure (OR 1.64, 95%CI 1.18–3.27, *p* = 0.003), but it did not reach significance as predictive factor of Δ eGFR loss >30% at last follow-up (HR 1.63, 95%CI 0.87–5.71, *p* = 0.11). During Cox regression analysis to explore the clinical predictors independently associated with tumor recurrence, the pathological nucleolar grade showed a significative correlation (HR 1.14, 95%CI 1.05–1.21, *p* = 0.01).

## 4. Discussion

The management of renal tumors in patients with a solitary kidney presents unique challenges, as preserving renal function and ensuring effective cancer control must both be prioritized. Different therapeutic strategies (cryoblation, partial nephrectomy, active surveillance) can be choose on a case-by-case basis. However, surgical removal of the renal tumor remains the gold standard according to international guidelines [16]. This single-center prospective study adds to the current knowledge by evaluating perioperative, oncological and functional outcomes of robot-assisted partial nephrectomy (RAPN) in 39 patients with solitary kidneys.

Functional outcomes are a pivotal consideration in solitary-kidney patients [17]. The median percentage of eGFR loss from baseline to the third postoperative day and to last follow-up were 20% (IQR 5–45) and 10% (IQR 7–30), indicating a good recovery of renal function during the follow-up in this patient setting. Notably, 13 (33.3%) and only 7 (17.9%) patients had a eGFR loss > 30% at the third postoperative day and at the last follow-up, respectively.

We showed that the type of ischemia represents the prominent modifiable factor influencing renal function loss after surgery. The surgical decision-making process for clamping strategy includes parameters such us tumor location and dimension, as well as kidney and tumor vascularization. Notably, 25.6% of patients received selective clamping and 20.6% of procedures were completed without clamping, demonstrating a strong commitment to minimizing tissue damage in the solitary kidney. Certainly, finding the optimal balance between reducing bleeding risk during tumor removal—significantly decreased by global clamping—and minimizing the risk of incomplete tumor resection with positive surgical margins remains crucial [18,19]. From this perspective, the pneumoperitoneum pressure utilized in robotic surgery can help reduce venous bleeding at the resection site. Additionally, the enhanced visualization provided by the robotic system may further decrease the likelihood of positive margins. In this specific patient group, an enucleation resection technique, employed at our institution for many years, may not only lower positive surgical margins but also maximize healthy tissue preservation and thus limit blood loss. Our findings align closely with other studies that highlight the critical importance of preserving kidney tissue volume to optimize long-term kidney function in patients with solitary kidneys undergoing partial nephrectomy [20,21].

Functional preservation is critical for solitary-kidney patients, where even minor declines in renal function may significantly impact the patient’s quality of life. In our analysis, the median percentage of eGFR loss from baseline was 20% at the third postoperative day and decreased further to 10% at the last follow-up, indicating favorable renal recovery over time, even in this challenging patient group. Notably, only a minority of patients experienced substantial renal function impairment. Physical status (ASA score > II) emerged as the most influential predictor of significant long-term renal function loss, underscoring the crucial role of patient-related factors in predicting outcomes. While global ischemic clamping was significantly associated with early postoperative renal impairment and Trifecta failure, it did not significantly predict long-term renal function loss. This finding aligns with the recent literature, emphasizing that ischemic clamping predominantly influences short-term outcomes, whereas long-term renal function primarily depends on nonmodifiable factors such as baseline renal function and preserved parenchymal volume [21].

Positive surgical margins (PSMs) were detected in 15.3% of cases, which is higher than reported in some previous series [21]. We believe that the major determinant was the fact that, whenever feasible, these procedures were conducted in an off-clamp fashion. While PSMs do not invariably lead to recurrence, they warrant careful intraoperative planning and consideration of frozen section analysis to enhance oncological outcomes [22,23].

The achievement of Trifecta, defined by the absence of major complications (Clavien-Dindo > 2), negative surgical margins and a less than 30% decline in eGFR, was accomplished in 66.6% of cases. These results align with previously reported success rates of RAPN in solitary-kidney patients, further substantiating its role as a feasible nephron-sparing surgical option for such complex cases [24,25]. However, it is noteworthy that global clamping and ASA scores >2 emerged as independent predictors of Trifecta failure. Selective or zero-ischemia strategies, when feasible, may mitigate ischemic damage, improving functional outcomes without compromising oncological control [26]. In this light, recent technological innovations, which can be integrated into the robotic console, may assist the surgeon in the preoperative and intraoperative phases, leading to a more accurate tailoring of the surgical strategy [27,28,29].

From an oncological perspective, this study confirms the high efficacy of RAPN. A majority of cases (84.5%) were stage cT1, with clear cell renal cell carcinoma (RCC) being the predominant histotype. Despite the advanced disease observed in 17.6% of cases with definitive pathology (pT3a and pT3b), the cancer-specific survival rate reached 100% at a median follow-up of 28 months, which is consistent with favorable outcomes reported in the literature for minimally invasive partial nephrectomy [30,31]. The systemic recurrence rate (20.5%) and local recurrence rate (10.2%) observed in the study emphasize the need for stringent postoperative surveillance protocols, particularly in patients with adverse pathological features such as sarcomatoid differentiation and tumor necrosis. We speculated that this relatively high recurrence rate could be due to the fact that more than half of the patients had a single kidney due to a previous RCC history, and so the dissemination of the disease had probably already started at the moment of the diagnosis. Moreover, the systemic nature of several of the reported recurrences highlights the pivotal role of nephron-sparing surgery in this patient setting [32]. Indeed, renal function preservation represents a key element in patients with higher risk of recurrence to allow for medical management in both the adjuvant and the metastatic setting and improve the overall survival rates, according to a growing body of evidence [33]. To date, in our study, the presence of a nucleolar grade > 2 was the only confirmed predictor of disease recurrence, thus suggesting the importance of embracing a tailored follow-up schedule in patients with a higher risk of recurrence.

The limitations of the study should be acknowledged. First, the single-center design and the relatively small sample size may limit the reliability of the reported results; nevertheless, all cases were managed in a high-volume tertiary referral center, and the presence of RCC-harboring solitary-kidney cases represents a rare event. Additionally, the short- to mid-term follow-up precludes a comprehensive evaluation of long-term outcomes, particularly regarding renal function and late recurrence. Moreover, the absence of genetic testing in this particular patient setting might hide several important factors, particularly those regarding the disease recurrence analyses. The lack of a comparative cohort focused on ablative techniques is also a limitation. However, we believe that this study represents a valuable piece of evidence supporting RAPN in single-kidney patients, demonstrating that, in a referral center and through a tailored surgical strategy, this therapeutic option can be pursued while maintaining high standards of care. Moreover, the centralization of such patients in referral robotic centers would strengthen the health economics perspective, also considering that novel technologies, including three-dimensional reconstruction, can further increase the chance of reaching Trifecta [34,35,36].

## 5. Conclusions

Robot-assisted partial nephrectomy led to valuble functional and oncological outcomes in patients with solitary kidneys. In this context, the identification of clinical predictors of Trifecta failure, such as global clamping and ASA score > II, allows for the optimization of the patient selection process and of surgical planning. Future multicenter and technology-integrated studies with larger cohorts and longer follow-up periods are essential to validate these findings and refine the management of this high-risk population.

## Figures and Tables

**Table 1:** Preoperative patient- and tumor-related features in the overall cohort.

		Overall Number (n = 39)	%/IQR
**Age, years—median (IQR)**		65	56–71
**Sex—n. (%)**	**Male**	28	71.8
	**Female**	11	28.2
**Side—n. (%)**	**Right**	19	48.7
	**Left**	20	51.3
**BMI, kg/m^2^—median (IQR)**		27.2	25.8–30.9
**ASA score—n. (%)**	**1**	4	10.2
	**2**	20	51.2
	**3**	15	38.6
**CCI—median (IQR)**		2	4–5
**Baseline creatinine (mg/dL)—median (IQR)**		1.5	1.0—2.0
**Baseline eGFR (mL/min/1.73 m²)—median (IQR)**		45	35–80
**Baseline chronic kidney disease stage—n. (%)**	**1**	0	0
	**2**	11	28.2
	**3a**	9	23.1
	**3b**	14	35.9
	**4**	5	12.8
**Single kidney reason—n. (%)**	**Surgical**	21	53.8
	**Congenital**	10	25.6
	**Functional**	8	20.6
**PADUA score, median (IQR)**		7	8–9
**RENAL score, median (IQR)**		5	6–7
**Tumor diameter, mm—median (IQR)**		32	21–49
**MAP-score, n. (%)**	**0**	10	25.6
	**1**	7	18.0
	**2**	11	28.2
	**3**	6	15.3
	**4**	3	7.6
	**5**	2	5.3
**Clinical T-stage, n. (%)**	**1a**	24	61.5
	**1b**	9	23.0
	**2**	5	13.8
	**3a**	0	0
	**3b**	1	2.7

ASA: American Society of Anesthesiologists; BMI: body mass index; CCI: Charlson comorbidity index; IQR: interquartile range; MAP: Mayo adhesive probability; mm: millimeters; PADUA: preoperative aspects and dimensions used for anatomy; RENAL: radius–exophycity–nearness–anterior–location.

**Table 2:** Perioperative surgical outcomes in the overall cohort.

		Overall Number (n = 39)	%/IQR
**Surgical access, n. (%)**	**Transperitoneal**	31	79.4
	**Retroperitoneal**	8	20.6
**Operative time, minutes—median (IQR)**		87	115–160
**Clamping—n. (%)**	**Global**	21	53.8
	**Selective**	10	25.6
	**No clamp**	8	20.6
**Ischemia time, minutes—median (IQR)**		12	10–18
**Intraoperative complication, n. (%)**		0	0
**EBL, mL—median (IQR)**		300	250–450
**Postoperative complication—n. (%)**		25	64.0
**CD classification—n. (%)**	**I**	9	23.1
	**II**	12	30.7
	**III**	3	7.6
	**IV**	1	2.6
**Hospital stay, days—median (IQR)**		4	3–5
**Drainage time, days—median (IQR)**		2	2–3
**Δ Hemoglobin blood level, g/dL—median (IQR)**		−2.9	−2.2–−3.5
**Δ Creatinine serum level at 3rd postoperative day, mg/dL—median (IQR)**		1.5	1.1–1.9
**Δ eGFR serum level at 3rd postoperative day, mg/dL—median (IQR)**		20	5–45
**Positive surgical margin, n. (%)**		6	15.3
**Trifecta rate, n. (%)**		26	66.6

CD: Clavien–Dindo; EBL: estimated blood loss; eGFR: estimated glomerular function rate; Δ: delta (difference); IQR: interquartile range.

**Table 3:** Postoperative pathological outcomes in the overall cohort.

		Overall Number (n = 39)	%/IQR
**Malignant histotype—n. (%)**	**Clear cell RCC**	27	69.2
	**Papillary RCC**	2	5.1
	**Chromophobe RCC**	2	5.1
	**Other malignant histotype**	3	7.9
**Benign histotype—n. (%)**	**Oncocytoma**	4	10.2
	**Angiomyolipoma**	1	2.5
	**Other benign histotype**	0	0
**Pathological tumor diameter, mm—median (IQR)**		30	20–49
**Nucleolar grade—median (IQR)**	**I**	4	11.7
	**II**	16	47.1
	**III**	9	26.4
	**IV**	5	14.8
**Pathological T-stage—n. (%)**	**1a**	16	47.2
	**1b**	10	29.3
	**2**	2	5.9
	**3a**	5	14.7
	**3b**	1	2.9
**Presence of tumor necrosis, n. (%)**		3	8.8
**Sarcomatoid differentiation, n (%)**		3	8.8

IQR: interquartile range; mm: millimeters; RCC: renal cell carcinoma; T: tumor.

**Table 4:** Clinical and surgical data of patients reaching vs. not reaching Trifecta.

		Patients Reaching Trifecta (n = 26)	Patients Not Reaching Trifecta (n = 13)	*p*-Value
**Age, years—median (IQR)**		65 (56–71)	67 (58–71)	0.18
**ASA score, n. (%)**	**1**	8 (30.7)	1 (7.6)	<0.001
	**2**	14 (53.8)	3 (23.0)	
	**3**	4 (15.5)	9 (69.4)	
**Clinical tumor diameter (mm), median (IQR)**		30 (21–47)	32 (22–49)	0.07
**PADUA score, median (IQR)**		7 (8–9)	7 (8–10)	0.18
**Clinical T/stage, n. (%)**	**1a**	18 (69.2)	6 (46.1)	0.09
	**1b**	5 (19.2)	4 (30.7)	
	**2**	2 (7.6)	3 (23.2)	
	**3a**	0 (0.0)	0 (0.0)	
	**3b**	1 (4.0)	0 (0.0)	
**Operative time, minutes—median (IQR)**		85 (111–159)	89 (118–162)	0.31
**MAP-score, n. (%)**	**0**	5	5	0.32
	**1**	3	4	
	**2**	6	5	
	**3**	3	3	
	**4**	2	1	
	**5**	0	2	
**Clamping, n. (%)**	**Global**	9 (34.6)	12 (92.3)	<0.001
	**Selective**	9 (34.6)	1 (7.7)	
	**No clamp**	8 (30.8)	0 (0.0)	
**EBL, mL—median (IQR)**		350 (300–450)	250 (300–350)	0.06

ASA: American Society of Anesthesiologists; EBL: estimated blood loss; MAP: Mayo adhesive probability; PADUA: preoperative aspects and dimensions used for anatomy.

**Table 5:** Multivariate logistic regression analyses for Δ% eGFR loss > 30% at the third postoperative day, Trifecta failure and Δ% eGFR loss > 30% at last follow-up.

	(% eGFR Loss > 30% at 3rd POD	Trifecta Failure	(% eGFR Loss > 30% at Last Follow-Up	Disease Recurrence
	OR, (95% CI), p	OR, (95% CI), p	HR, (95% CI), p	HR, (95% CI), p
ASA score (>II vs. ≤II)	1.19, (1.13–4.21), 0.002	1.24, (1.10–3.87), 0.001	2.21, (1.47–6.81), <0.0001	-
Global clamping (vs. selective/clampless)	1.82, (1.43–2.31), <0.0001	1.64, (1.18–3.27), 0.003	1.63, (0.87–5.71), 0.11	-
Nucleolar grade > 2	-	-	-	1.14, (1.05–1.21), 0.01

ASA: American Society of Anesthesiologists; OR: odds ratio; HR: hazard ratio; CI: confidence interval; POD: postoperative day.

## Data Availability

All procedures described in this study involving human participants were performed in accordance with the ethical standards of the institutional and national research committee and with the 1964 Helsinki Declaration and its later amendments or comparable ethical standards.

## Supplementary Materials

The following supporting information can be downloaded at: https://www.mdpi.com/article/10.3390/cancers17121978/s1, Table S1: Recurrence and complication rates stratified by tumor stage and clamping type.

## Abbreviations

The following abbreviations are used in this manuscript:

RAPN	Robot-Assisted Partial Nephrectomy
NSS	Nephron-Sparing Surgery
RCC	Renal Cell Carcinoma
BMI	Body Mass Index
CCI	Charlson Comorbidity Index
ASA	American Society of Anesthesiologists
CT	Computed Tomography
MRI	Magnetic Resonance Imaging
PADUA	Preoperative Aspects and Dimensions Used for Anatomy
RENAL	Radius–Exophycity–Nearness–Anterior–Location
CD	Clavien–Dindo
EAU	European Association of Urology
LR	Local Recurrence
SR	Systemic Recurrence
EGFR	Estimated Glomerular Filtration Rate
IQR	Interquartile Range
ISUP	International Society of Urological Pathology
PSMs	Positive Surgical Margins
MM	Millimeters
EBL	Estimated Blood Loss
OD	Odds Ratio
HR	Hazard Ratio
CI	Confidence Interval
POD	Postoperative Day
MAP	Mayo Adhesive Probability
