# Supplementary material for: Functional and Oncologic Outcomes in Single-Kidney Patients Treated with Robot-Assisted Partial Nephrectomy for Renal Tumors: Results from a Prospectively Maintained Dataset of a Single Tertiary Referral Center
Source: Cancers (Basel). 2025 Jun 13;17(12):1978. doi: 10.3390/cancers17121978 (PMC12190960; doi:10.3390/cancers17121978)
Supplement: Supplementary file 1 [file cancers-17-01978-s001.zip › cancers-3634292-supplementary.pdf]

Supplementary Table S1. Recurrence and complication rates stratified by tumor stage and clamping type

		Patients free from disease (n=27)	Patients with tumors recurrence (n=12)	p-value
**Age, years – median (IQR)**		65 (56 – 70)	67 (56 – 71)	0.18
**PADUA score, median (IQR)**		7 (8 - 9)	7 (8 – 9)	0.24
**RENAL score, median (IQR)**		5 (6 – 7)	5 (6 – 7)	0.42
**Pathological T-stage, n. (%)**	**1a**	22 (56.4)	2 (5.1)	**0.02**
	**1b**	4 (10.2)	5 (12.8)	
	**2**	1 (2.5)	4 (10.2)	
	**3a**	0 (0.0)	0 (0.0)	
	**3b**	0 (0.0)	1 (2.5)	
**Clamping, n. (%)**	**Global**	15 (38.4)	6 (15.3)	0.09
	**Selective**	5 (12.8)	5 (12.8)	
	**No-clamp**	7 (17.9)	1 (2.5)	
		**Patients not experiencing complication (n=14)**	**Patients experiencing complication (n=25)**	**p-value**
**Age, years – median (IQR)**		65 (56 – 70)	67 (59 – 71)	0.14
**PADUA score, median (IQR)**		6 (8 - 9)	7 (8 – 9)	0.28
**RENAL score, median (IQR)**		5 (6 – 7)	5 (6 – 7)	0.31
**Pathological T-stage, n. (%)**	**1a**	11	13	**0.02**
	**1b**	2	7	
	**2**	1	4	
	**3a**	0	0	
	**3b**	0	1	
**Clamping, n. (%)**	**Global**	8 (20.5)	13 (33.3)	0.06
	**Selective**	4 (10.2)	6 (15.3)	
	**No-clamp**	2 (5.1)	6 (15.3)	
